# Allopregnanolone Promotes Regeneration and Reduces β-Amyloid Burden in a Preclinical Model of Alzheimer's Disease

**DOI:** 10.1371/journal.pone.0024293

**Published:** 2011-08-30

**Authors:** Shuhua Chen, Jun Ming Wang, Ronald W. Irwin, Jia Yao, Lifei Liu, Roberta Diaz Brinton

**Affiliations:** 1 Department of Pharmacology and Pharmaceutical Science, School of Pharmacy, University of Southern California, Los Angeles, California, United States of America; 2 Department of Pathology, University of Mississippi Medical Center, Jackson, Mississippi, United States of America; 3 Neuroscience Program, University of Southern California, Los Angeles, California, United States of America; Hokkaido University, Japan

## Abstract

Previously, we demonstrated that allopregnanolone (APα) promoted proliferation of rodent and human neural progenitor cells *in vitro.* Further, we demonstrated that APα promoted neurogenesis in the hippocampal subgranular zone (SGZ) and reversed learning and memory deficits in the male triple transgenic mouse model of Alzheimer's (3xTgAD). In the current study, we determined the efficacy of APα to promote the survival of newly generated neural cells while simultaneously reducing Alzheimer's disease (AD) pathology in the 3xTgAD male mouse model. Comparative analyses between three different APα treatment regimens indicated that APα administered 1/week for 6 months was maximally efficacious for simultaneous promotion of neurogenesis and survival of newly generated cells and reduction of AD pathology. We further investigated the efficacy of APα to impact Aβ burden. Treatment was initiated either prior to or post intraneuronal Aβ accumulation. Results indicated that APα administered 1/week for 6 months significantly increased survival of newly generated neurons and simultaneously reduced Aβ pathology with greatest efficacy in the pre-pathology treatment group. APα significantly reduced Aβ generation in hippocampus, cortex, and amygdala, which was paralleled by decreased expression of Aβ-binding-alcohol-dehydrogenase. In addition, APα significantly reduced microglia activation as indicated by reduced expression of OX42 while increasing CNPase, an oligodendrocyte myelin marker. Mechanistic analyses indicated that pre-pathology treatment with APα increased expression of liver-X-receptor, pregnane-X-receptor, and 3-hydroxy-3-methyl-glutaryl-CoA-reductase (HMG-CoA-R), three proteins that regulate cholesterol homeostasis and clearance from brain. Together these findings provide preclinical evidence for the optimal treatment regimen of APα to achieve efficacy as a disease modifying therapeutic to promote regeneration while simultaneously decreasing the pathology associated with Alzheimer's disease.

## Introduction

Alzheimer's disease is the result of a multifactorial disease process that ultimately leads to a decline in neural plasticity, neuroregenerative capacity, and development of amyloid-beta (Aβ) plaques and neurofibrillary tangles [1]. In addition, Alzheimer's disease is also associated with myelination abnormalities in specific brain regions that are most vulnerable to AD pathology, including the hippocampus and entorhinal cortex [2]. Studies of quantitative volumetric magnetic resonance imaging assessments have revealed white matter atrophy within these regions in brains of incipient stage AD patients [3]–[4].

The triple transgenic mouse model of Alzheimer's disease (3xTgAD) mouse developed by Oddo, LaFerla and colleagues bears mutations in three genes (human APP_SWE_, Tau_P301L_, and PS1_M146V_ genes) linked to AD and fronto-temporal dementia and exhibits an age-related neuropathological phenotype including both Aβ deposition and tau hyperphosphorylation [5]. Further, similar to human AD progression, 3xTgAD mice exhibit significant region-specific alterations in myelination and oligodendrocyte profile prior to the development of Aβ and tau pathology [6].

Previously, we demonstrated that allopregnanolone (APα, 3α-hydroxy-5α-pregnan-20-one) increased proliferation of neural progenitor cells *in vitro* [7]. Further, acute single administration of APα reversed both neurogenic and cognitive deficits *in vivo* in male 3xTgAD mice prior to the appearance of AD pathology [8]. We further demonstrated that APα successfully promoted neurogenesis and reversed cognitive deficits in male 3xTgAD mice following the onset of AD pathology. Humans with AD display reduced levels of cortical APα, which were inversely correlated with Braak and Braak neuropathological disease stage [9], [10]. The APOE4 allele is also associated with reduced APα levels [10]. Aside from Alzheimer's, APα has also been shown to increase myelin basic protein in organotypic slice cultures of rat cerebellum [11] and delay demyelination in Niemann-Pick C mice [12]. The mechanism of APα induced protection of myelin integrity is suggested to involve liver X receptor (LXR) and pregnane X receptor (PXR) systems, important regulators of cholesterol, fatty acid, and glucose homeostasis [13], [14]. Interestingly, the LXR and PXR synthetic ligand, T0901317 significantly decreased Aβ secretion and increased the expression of ABCA1, an enzyme involved in Aβ clearance, in 11-week-old APP23 mice [15].

In this study, we first determined the optimal APα treatment regimen to achieve regenerative benefits and reduction of AD pathology in the male 3xTgAD mouse model. Comparative analyses between three different APα treatment regimens indicated that APα administered 1/week for 6 months was maximally efficacious for simultaneous promotion of neurogenesis and survival of newly generated cells and reduction of AD pathology. We further investigated the impact of different therapeutic intervention stages (pre- and post- Aβ pathology) of the 1/week/6 months APα treatment to reduce AD pathology and preserve myelination.

## Results

### Determination of the Optimal APα Treatment Regimen to Promote Regenerative Capacity and Reduce β- Amyloid

**Figure 1:**
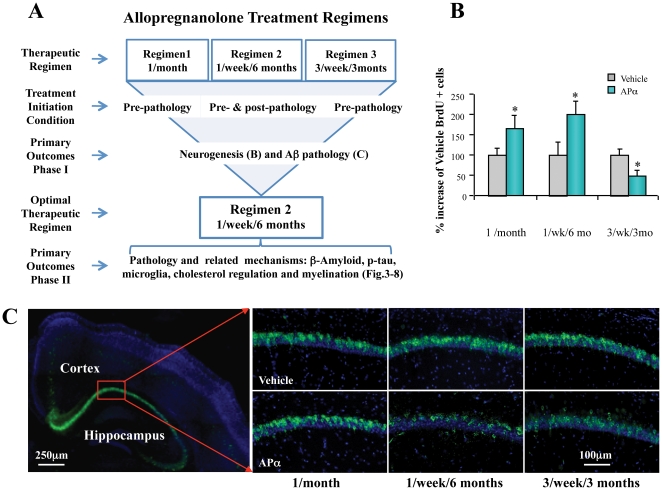
Determination of optimal allopregnanolone treatment regimen. (A). Schematic overview of three APα treatment designs and their outcomes. (B) Impact of APα on neurogenesis in 3xTgAD mouse model. All three APα treatments were initiated when mice were 3 months old. Upon completion of each treatment paradigm, BrdU stereology (for 3/week/3 months treatment paradigm, *n* = 7–8/group) or flow cytometry assay (for 1/week/6 months treatment paradigm, *n* = 2–5/group and 1/month treatment paradigm, *n* = 10–12/group) was used to assess neurogenesis. Both the 1/month APα treatment and the 1/week/6 months APα treatment induced a significant increase in neurogenesis, with the latter regimen yielding the greater increase in neurogenesis. However, the 3/week/6 months treatment induced a significant decrease in neurogenesis. The percentage of increase is presented as mean ± SEM * *P*<0.01. (C) Impact of APα on Aβ accumulation in 3xTgAD mouse model. Brain sections from 3xTgAD mice treated with APα (10 mg/kg) or vehicle were stained. Aβ immunoreactivity was detected with 6E10 antibody (green) and nuclei counter-stained with DAPI (blue). Representative images indicated that the 1/week/6 months APα treatment significantly decreased 6E10 immunoreactivity and showed the highest efficacy of Aβ reduction; whereas the 3/week/3 months APα treatment had a comparable efficacy of Aβ reduction and the 1/month APα treatment showed minimal effect of reducing Aβ immunoreactivity.

Our previous studies indicated dose-dependent efficacy of APα on neurogenesis [7], [8]. To determine the optimal regimen for therapeutic efficacy of APα treatment, we investigated the efficacy of three APα treatment paradigms depicted in Figure 1A, which were designed to determine the optimal APα treatment regimen to both promote the regenerative capacity of the brain and to reduce Aβ pathology. Three different APα treatment regimens were compared. The 1/month single injection of APα treatment regimen replicated our previous approach [8] whereas the chronic treatment 3/week/3 months and 1/week/6 months paradigms were developed to simulate potential clinical treatment regimens. The optimal dose of 10 mg/kg APα was based on previous analyses [8] and was continued in this study for all three treatment regimens. Both APα treatment regimens of a single exposure of 1/month and 1/week/6 months APα treatment significantly increased the survival of cells that were generated at the first exposure to APα. The 1/week/6 months APα treatment regimen had greater regenerative efficacy (Fig. 1B, *P*<0.01). However, the 3/week/3 months regimen significantly reduced regenerative efficacy (Fig. 1B). In addition, we conducted pilot immunofluorescent labeling assays to assess the efficacy of different APα treatment regimens to reduce amyloid beta (Aβ) accumulation. Results indicated that both the 1/week/6 months regimen and the 3/week/3 months regimens significantly reduced Aβ accumulation in hippocampal CA1 pyramidal neurons, whereas the 1/month single dose of 10 mg/kg APα failed to reduce Aβ accumulation (Fig. 1C). Together these data suggest that the 1/week/6 months APα treatment regimen is optimal for APα to both achieve regenerative efficacy and reduce Aβ pathology.

### Window of Therapeutic Efficacy

Upon determination that the 1/week/6 months regimen was the optimal treatment paradigm as it induced the greatest efficacy for promoting regenerative capacity and reducing Aβ pathology, we expanded our investigation to explore the impact of initiating APα treatment at pre- versus post- Aβ pathology. Specifically, we initiated the 1/week/6 months APα treatment when mice were 3 months of age with no observable amyloid pathology or when mice were 6 months of age with overt intraneuronal accumulation of Aβ [5], [8]. Both treatment paradigms were for six months. Mice treated at 3 months of age were 9 months of age at the end of treatment when intraneuronal Aβ is apparent. Mice that began treatment at 6 months of age had intraneuronal Aβ and by 12 months of age at sacrifice would have developed Aβ plaques.

### Allopregnanolone Promoted Survival of Newly Generated Neural Cells

**Figure 2:**
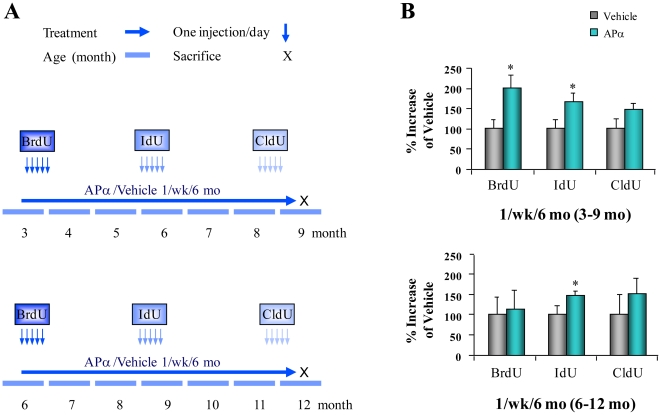
Impact of optimal 1/week/6 m allopregnanolone treatment regimen on neurogenesis and survival in 3xTgAD male mouse. (A) The paradigm of once per week for 6 months of treatment. Three-month-old (pre-detectable pathology) and six-month-old (readily detectable pathology) male 3xTgAD mice were randomized into vehicle or APα treatment groups. APα or vehicle was subcutaneously administered at 10 mg/kg once per week for 6 months (1/week/6 month). To assess the survival capability of newly generated cells, mice were treated with sequential injections of BrdU for the first 5 days of the study (indicating a 6 month survival period), IdU for 5 days at the midpoint (indicating a 3 month survival period), and CldU 5 days before completion of the study (indicating a short term survival period). After 6 months of APα treatment, mice at 9- and 12-months-old were sacrificed. (B) Impact of survival of newly generated cells in hippocampus subgranular zone (SGZ). Flow cytometry analysis showed APα treatment (1/week/6 months) significantly increased IdU positive cells in 9-month-old (upper panel) and 12-month-old mice (lower panel); BrdU positive cells were increased in 9-month-old mice (upper panel). CldU positive cells were not significantly increased in both ages suggesting that this APα treatment regimen sustained survival of newly generated cells, and that neurogenic capacity of APα decreased with age. The percentage increase was presented as mean ± SEM; * *P*<0.01; *n* = 2–5/group.

To further evaluate the short term, mid term, and long term survival of newly generated cells in the 1/week/6 months APα treatment paradigm, three thymidine analogs, (BrdU, IdU and CldU) were injected at the beginning (BrdU), middle (IdU) and end (CldU) of the experiment following injections of APα to distinguish the survival of newly generated cells for the duration of 6 months (BrdU+), 3 months (IdU+) and one week (CldU+) (Fig. 2A). Results from flow cytometry analysis indicated that APα treatment of 1/week/6 months initiated at 3 months of age significantly increased the long term (6 months; BrdU+, 98.45% ±22.92) and mid term (3 months; IdU+, 68.77%±18.68) survival of newly generated cells and moderately increased the short term (1 week; CldU+, 48.36%±15.42) survival of newly generated cells (Fig. 2B upper panel). The population of IdU positive cells was also increased by APα treatment initiated at 6 months of age.

### Allopregnanolone Reduced Aβ Oligomer Accumulation

**Figure 3:**
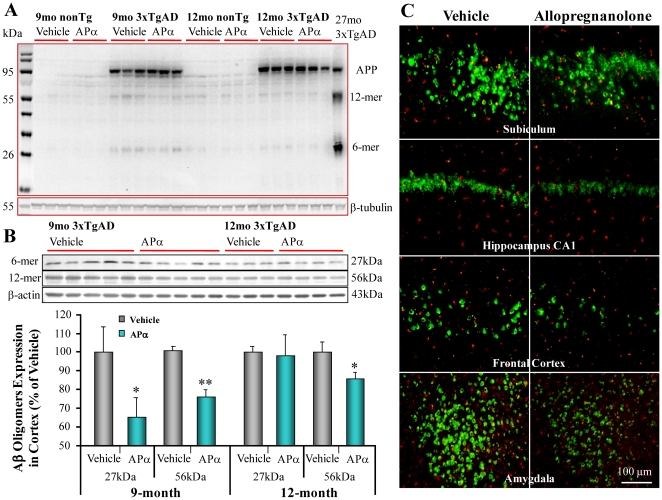
Allopregnanolone reduced expression of Aβ oligomers in 9- and 12-month-old male 3xTgAD mice. Equal amounts of frontal-parietal-temporal cortex samples from 3xTgAD mice treated with APα (10 mg/kg) or vehicle were loaded onto the gel. Aβ expression was determined with Aβ antibody 6E10 by immunoblot analysis. (A) Three major immunoreactive bands were detected in the samples from 9 and 12 month old 3xTgAD mouse brains. Full-length APP band was detected at ∼100 kD. The bands at ∼27 kD and 56 kD indicated Aβ hexamers (6-mer) and Aβ dodecamers (12-mer) respectively. One 27-month old 3xTgAD cortex sample was included as a positive control. (B) For better separation of the Aβ 56 and 27 kD bands, a 10–20% gradient gel was used to analyze the effects of APα on Aβ oligomers. APα treatment significantly reduced Aβ 56 kD oligomer in the pre-pathology treatment (25±4%, *n* = 5, *P*<0.01) and the post-pathology treatment (15±4%, *n* = 3–4, *P* = 0.05) groups. Aβ 6-mer at ∼27 kDa was also significantly reduced by APα treatment in the pre-pathology treatment group (35±10%, *P*<0.05). Bars represent mean relative expression ± SEM (* *P*≤0.05 and ** *P*<0.01 compared with vehicle control group). (C) Region specific reduction in Aβ IR by APα treatment in 3xTgAD brains. Representative images of Aβ immunostaining indicated a significant decrease of Aβ IR in the hippocampus and amygdala in APα-treated 3xTgAD mice relative to vehicle.

The 3xTgAD mouse model used in the current study exhibits an age-related neuropathological progression pattern [5]. Analyses of pathological AD phenotype confirmed that mice derived from our colony expressed age-dependent and region specific Aβ and tau pathology (data not shown). We first characterized the expression of different forms of Aβ in 3xTgAD mice. Western blot analyses of cortex from 3xTgAD mice revealed three major Aβ-related bands: full-length APP band at ∼100 kD; oligomeric Aβ bands at ∼27 kD and ∼56 kD representing hexamers (6-mer) and dodecamers (12-mer) respectively (Fig. 3A). No immunoreactive bands were detected in samples derived from non-transgenic (nonTg) mice (Fig. 3A). One 27-month 3xTgAD brain sample was included as a positive control and confirmed an age-dependent increase in band intensity (Fig. 3A). Because Aβ 56 kD oligomer has been proposed to be responsible for cognitive and memory deficits in AD, our analyses focused on this oligomer and its precursor Aβ 27 kD. The 56 kD Aβ oligomer is likely derived from dimerization of the 27 kD Aβ oligomers, which requires both the availability of the 27 kD Aβ oligomer and the catalytic process of dimerization. Compared to vehicle treatment, APα treatment partially reduced Aβ*56 in both age groups, with a 25±4% reduction in the pre-pathology treatment group (*n* = 5, *P*<0.01) and 15±4% reduction in the post-pathology treatment group (*n* = 3–4, *P* = 0.05). APα treatment also reduced the level of Aβ 6-mer as indicated by a significant reduction in 27 kD band (35±10%, *n* = 5, *P*<0.05) intensity in the pre-pathology treatment group. In contrast, in the post-pathology treatment group, no significant reduction of Aβ 6-mer occurred (Fig. 3B). These data indicate that pre-pathology APα treatment is reducing the generation of the early oligomer (27 kD) and the late oligomer (56 kD) whereas the post-pathology APα treatment appears to selectively delay the formation of the 56 kD oligomer. In addition, APα had no effect on APP protein expression, suggesting that APα did not directly affect APP generation. Together these data suggest that APα reduced the oligomerization of Aβ. Additional immunofluorescent analyses confirmed the findings from Western blot that APα treatment induced an apparent reduction of Aβ immunoreactivity in specific brain regions including hippocampus, cortex, and amygdala (Figure 3C).

### Allopregnanolone Reduced ABAD Expression in 3xTgAD Mice

**Figure 4:**
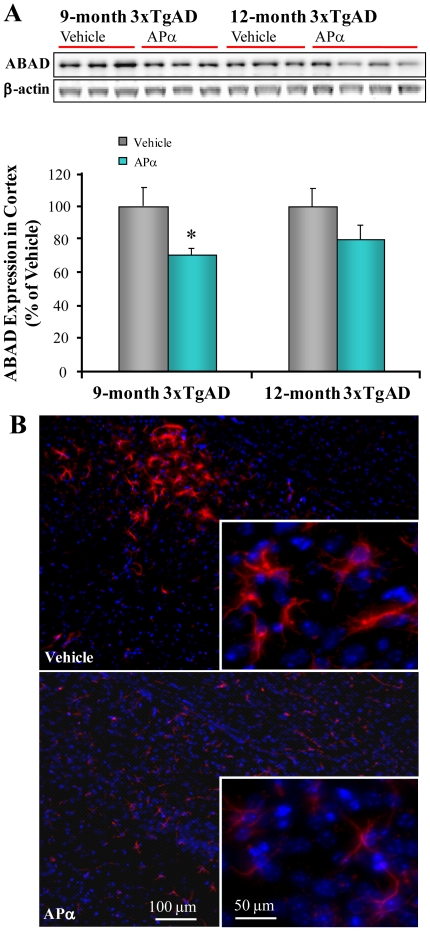
Allopregnanolone reduced ABAD expression in 3xTgAD mice. Equal amounts of frontal-parietal-temporal cortex samples from 3xTgAD mice treated with APα (10 mg/kg) or vehicle were loaded onto the gel. Aβ-binding alcohol dehydrogenase (ABAD) was probed with antibody ERAB (1∶500, Abcam) by immunoblot analysis. (A) APα-treatment significantly decreased ABAD levels in both the pre-pathology group (30±4%, *n* = 3, *P*<0.05) and the post-pathology treatment group (20±7%, *n* = 3–4, *P* = 0.07). Bars represent mean relative expression ± SEM; * *P*<0.05 compared with vehicle control group. (B) Confirmation of reduced ABAD expression by immunohistochemistry. Representative image of ABAD immunostaining in 3xTgAD mouse brain sections confirmed the results from immunoblots. There were much less ABAD immunoreactive cells seen in APα-treated brains relative to vehicle-treated brains.

The mitochondrial protein ABAD (amyloid-binding alcohol dehydrogenase) is over-expressed in human AD brains [16], [17], [18] and 3xTgAD mice [19]. Aβ binds to ABAD and disrupts mitochondrial function, leading to the generation of reactive oxygen species and cellular oxidative damage [18]. In parallel to APα-induced reduction of Aβ generation, APα-treatment significantly decreased cortical expression of ABAD in the pre-pathology group (30±4%, *n* = 3, *P*<0.05) and induced a trend of reduction in the post-pathology treatment group (20±7%, *n* = 3-4, *P* = 0.07) (Figure 4A). Similarly, additional immunofluorescent analyses revealed a qualitative reduction in ABAD immunofluorescent intensity in APα-treated 3xTgAD mouse brain sections relative to vehicle-treated brains (Fig. 4B). Together, APα treatment induced a parallel reduction of Aβ and ABAD expression indicating a potential protective mechanism whereby APα prevents Aβ induced mitochondrial dysfunction [18].

### Allopregnanolone Modulated Phosphorylated-tau Expression in 3xTgAD mice

**Figure 5:**
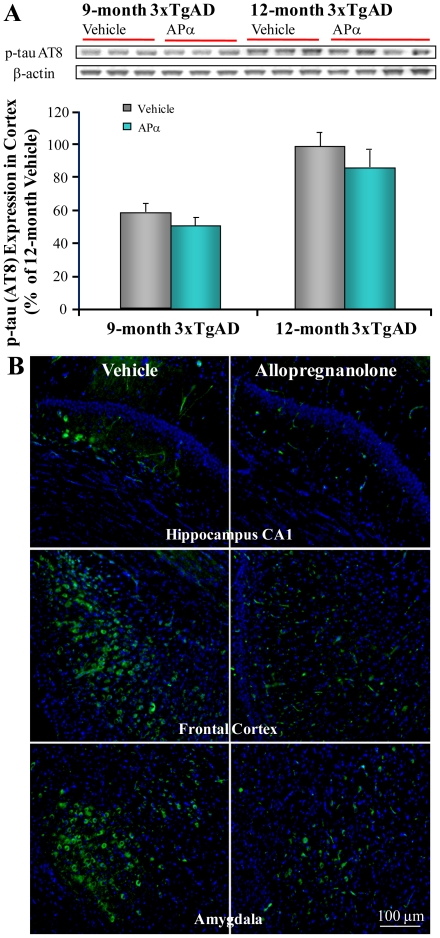
Allopregnanolone modulated phospho-tau expression in 3xTgAD mice. Equal amounts of frontal-parietal-temporal cortex samples from 3xTgAD mice treated with APα (10 mg/kg) or vehicle were loaded onto the gel. Phospho-tau expression was determined with phospho-tau antibody AT8 by immunoblot analysis. (A) No significant reduction of AT8 signal in APα-treated samples. Bars represent mean relative expression ± SEM. (B) Region-specific reduction of AT8 immunostaining in the hippocampus, cortex and amygdala in APα-treated mice. Representative image of AT8 immunostaining in 3xTgAD mouse brain sections indicated lower immunoreactivity in the hippocampus, cortex and amygdala of APα-treated mice relative to vehicle control.

In the 3xTgAD mouse model, tau pathology has been demonstrated to follow Aβ accumulation [20]. To determine whether the APα-induced reduction of Aβ levels would lead to reduction in tau pathology, we investigated the impact of APα on tau phosphorylation by Western blot analysis and immunofluorescent labeling with monoclonal phosphorylated-tau antibody (AT8), which recognizes phosphorylated tau serine 202. There was a trend towards reduction in phospho-tau band intensity in both pre-pathology treatment and post-pathology treatment groups, which did not reach statistical difference between APα- and vehicle-treated samples (Fig. 5A). However, immunofluorescent analyses indicated that APα induced a reduction in phospho-tau immunoreactivity at 12 months in the hippocampal CA1, frontal cortex and amygdalar regions (Figure 5B). In the pre-pathology treatment group when mice were at 9 months of age, phospho-tau was barely detectable.

### Allopregnanolone Regulated LXR, PXR and HMG-CoA-R Expression in 3xTgAD Mice

**Figure 6:**
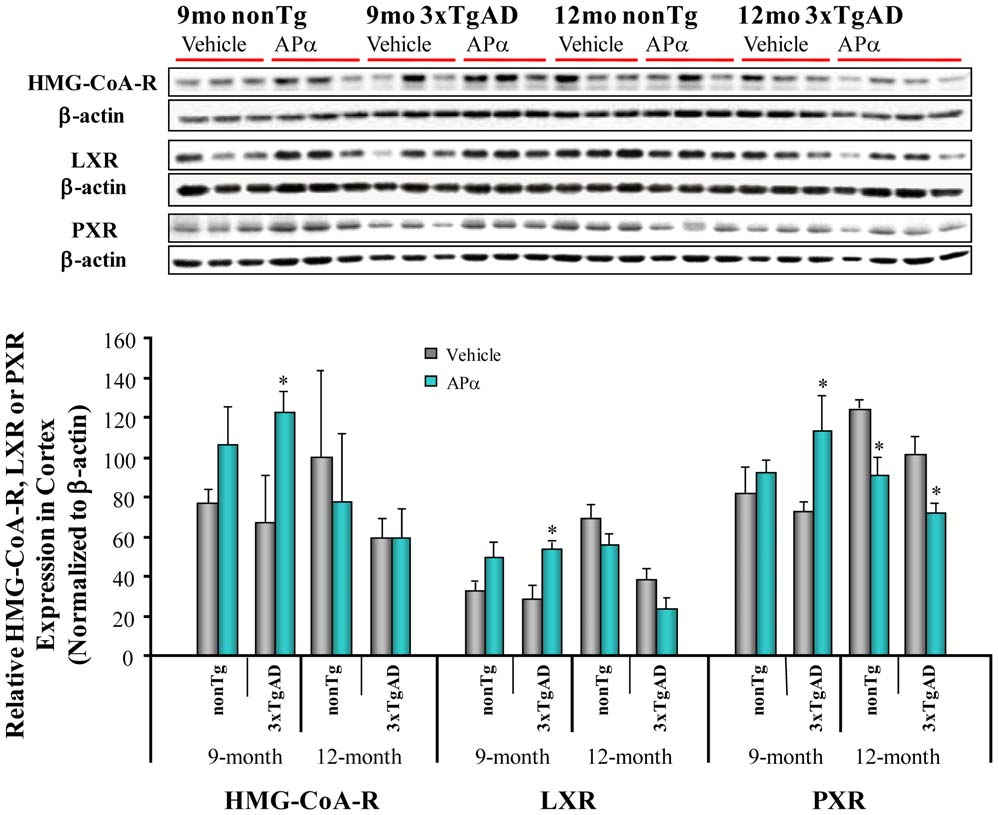
Allopregnanolone increased HMG-CoA-R, LXR and PXR expression in 9-month-old male 3xTgAD mice. Equal amounts of frontal-parietal-temporal cortex samples from 3xTgAD mice treated with APα (10 mg/kg) or vehicle were loaded onto the gel. Liver X Receptor (LXR), Pregnane X Receptor (PXR) or 3-Hydroxy-3-Methylglutaryl Coenzyme A Reductase (HMG-CoA-R), were determined with LXR, PXR, or HMG-CoA-R antibodies by immunoblot analysis. APα treatment increased the level of HMG-CoA-R (84%, *P* = 0.05), LXR (91%, *P*<0.05), and PXR (56%, *P*<0.05) in the pre-pathology treatment group. Bars represent mean relative expression ± SEM; * *P*≤0.05 compared with vehicle control group.

APα has been proposed to regulate cholesterol homeostasis via the LXR and PXR system [21], [22] and dysregulation of cholesterol homeostasis is associated with the generation of Aβ [15]. In the current study, we investigated APα regulation of inducible liver X receptor (LXR), pregnane X receptor (PXR) and 3-hydroxy-3-methylglutaryl coenzyme A reductase (HMG-CoA-R) expression. APα induced a significant increase in LXR, PXR and HMG-CoA-R expression in the pre-pathology treatment group. In contrast, in the post-pathology treatment group, APα treatment resulted in a reversal of cholesterol homeostatic responses, which was particularly evident in the significant reduction of PXR expression (Fig. 6). This suggests that the effect of APα is specific to the synthesis and clearance of cholesterol rather than the proteins involved in cholesterol trafficking.

### Allopregnanolone Treatment Inhibited Microglial Activation

**Figure 7:**
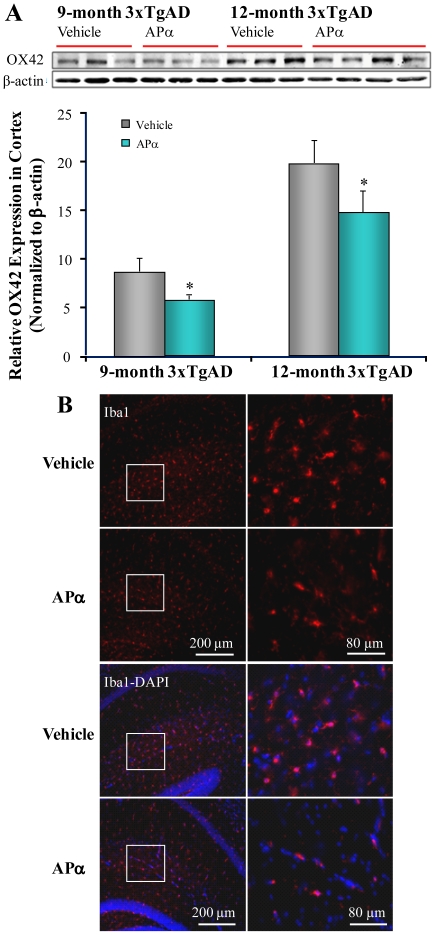
Allopregnanolone treatment inhibited microglial activation. Equal amounts of frontal-parietal-temporal cortex samples from 3xTgAD mice treated with APα (10 mg/kg) or vehicle were loaded onto the gel. Microglial activation was determined with anti-CD11b/c (OX42) antibody by immunoblot analysis. (A) APα treatment significantly decreased OX42 expression in both the pre-pathology treatment group (26.2±4.6%, *P*<0.05) and the post-pathology treatment group (18.0±7.4%, *P* = 0.05). Bars represent mean relative expression ± SEM; * *P*≤0.05. (B) Reduction in microglial staining by APα treatment in hippocampal CA1 region. Representative image of immunofluorescent staining with microglial marker anti-Iba1 in 3xTgAD brains indicated substantial reduction of reactive microglia in the hippocampal CA1 region in APα treated 3xTgAD mice relative to vehicle (Iba1-labeled microglia cells shown in red and DAPI-labeled nuclei shown in blue).

Microglial activation is well-documented in the pathogenesis of AD and is potentially related to presence of oligomerized Aβ. Further, in a model of cholesterol dysregulation, APα inhibited microglial activation in Niemann-Pick C mice [23]. The reduction in the 56 kD Aβ oligomer and ABAD in the post-pathology APα treatment group would be expected to result in a reduction of neuroinflammatory responses but to a lesser extent than the pre-pathology group. Consistent with these results, we observed a significant age-dependent increase in microglial activation in 3xTgAD mice as indicated by the significant increase activated microglia expression through immunoblotting with anti-CD11b/c (OX42) antibody, from 9 months to 12 months in the vehicle group mice (Fig. 7A, *P*<0.01). More importantly, compared to the vehicle control, APα treatment in 3xTgAD mice significantly decreased OX42 expression level in both the pre-pathology treatment (26.2±4.6%, *P*<0.05) and the post-pathology treatment group (18.0±7.4%, *P* = 0.05) (Fig.7A). Further immunofluorescent labeling with rabbit polyclonal anti-Iba1, another activated microglia marker, revealed APα treatment induced reduction of microglia immunoreactivity in hippocampal CA1 region (Fig. 7B).

### Allopregnanolone Increased a Marker of Myelination in Brains of 3xTgAD Mice

**Figure 8:**
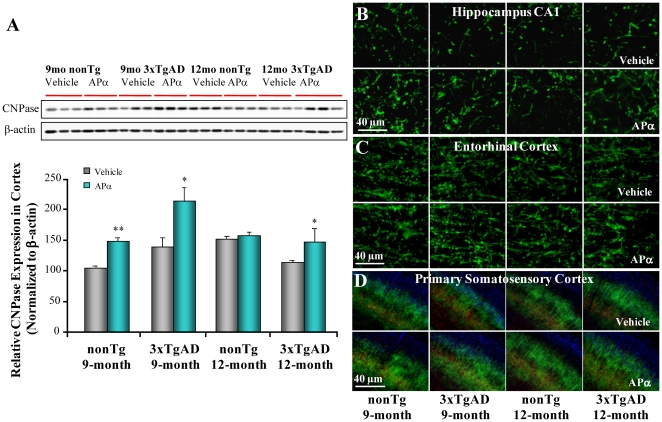
Allopregnanolone increased myelination in brain of 3xTgAD mice. Equal amount of frontal-parietal-temporal cortex samples from 3xTgAD mice treated with APα (10 mg/kg) or vehicle were loaded onto the gel. The expression of CNPase was measured using anti-CNPase antibody by immunoblot analysis as an indicator of oligodendrocytes and myelination. (A) APα treatment significantly increased CNPase expression in the pre-pathology group in both nonTg (*P*<0.01) and 3xTgAD mice (*P*<0.05). No significant change in CNPase expression was observed in the post-pathology treatment group in nonTg mice between APα treatment and the vehicle control. However, in 3xTgAD mice, APα treatment significantly increased CNPase expression (*P*<0.05). Bars represent mean relative expression values of CNPase relative to β-actin ± SEM; * *P*<0.05 and ** *P*<0.01 compared with vehicle control group. (B–D) Region-specific enhancement of myelination was observed in APα-treated mice. Representative image of CNPase immunostaining showed greater immunoreactivity in the hippocampal CA1 (B), entorhinal cortex (C) and primary somatosensory cortex (D) regions of APα-treated 3xTgAD mice.

Decline in myelination is a neuropathological consequence in the 3xTgAD animal model [6]. APα-enhanced myelination has been reported in Niemann-Pick C mice [23]. To investigate the therapeutic potential of APα to delay or reverse myelination deficits that occur in human AD [24], [25] and in 3xTgAD mice, we investigated the impact of APα on myelin expression in 3xTgAD mouse brain. APα induced a significant increase in the expression of CNPase, a marker of oligodendrocytes and myelin, relative to vehicle control by 40% in nonTg (*P*<0.01, *n* = 3) and 50% in 3xTgAD (*P*<0.05, *n* = 3) mice in the pre-pathology treatment group (Fig. 8A). Similarly, in the post-pathology treatment group, APα treatment also induced a 30% increase in CNPase levels in 3xTgAD mice (*P*<0.05, *n* = 3–4) (Fig. 8A). In the nonTg group, expression of CNPase increased in the 12-month-old vehicle group compared to 9-month-old vehicle, in which CNPase expression was not increased by APα (Fig. 8A). Immunofluorescent labeling with the same antibody revealed a region-specific increase in CNPase immunoreactivity in the hippocampal CA1 (B), entorhinal cortex (C) and primary somatosensory cortex (D) (Fig. 8B).

## Discussion

### Regenerative therapeutics that target AD pathology and the disease process

Previously we demonstrated that APα reversed neurogenic and cognitive deficits in the male 3xTgAD mouse model of Alzheimer's [8]. APα was efficacious in the 6- and 9-month old 3xTgAD mice on both neurogenesis and associative learning and memory and was comparable to their respective age-matched nonTg controls which corresponded to a 100% or more increase in response induced by APα [8], [26].

In vehicle-treated mice, there was a substantial age-related decline in neurogenesis that was exacerbated in 3xTgAD mice. Between 3 and 6 months there was a 78% decline and between 6 and 9 months there was another 58% decline, similarly an 18% decline was observed between 9 and 12 months [8], [26]. At 12 months of age in the 3xTgAD, APα no longer increased neurogenesis nor did it promote survival of neural progenitors. When extraneuronal Aβ plaques were present in the 12-month-old 3xTgAD mice, APα was ineffective in reversing the profound neurogenic and cognitive deficits of these mice. This is specific to the transgenic genotype because APα promoted neurogenesis and neuroprogenitor survival in the 15 month nonTg mice [26]. Treatment with APα increased neural progenitor survival and restored learning and memory to that of 12-month-old nonTg mice [26]. By the time 3xTgAD mice reached 12 months of age either the progenitor population was sufficiently depleted or the mechanisms by which APα promotes neuroproliferation and/or survival were no longer present and/or functional.

In the current study, we determined the efficacy of APα to promote the survival of newly generated neural cells while simultaneously reducing Alzheimer's disease (AD) pathology in the same mouse model with different APα treatment regimens. APα treatment paradigms included 1/month, 3/week/3 months, or 1/week/6 months, and were developed to simulate potential clinical regimens. The 1/month APα treatment regimen was efficacious in promoting neurogenesis and reversing cognitive deficits whereas it had no effect on AD pathology. In contrast, the most frequent treatment paradigm, 3/week/3 months APα was most efficacious for reducing AD pathology whereas it was least effective at promoting neurogenesis. Subsequent analyses indicated that APα (10 mg/kg) administered 1/week/6 months achieved an optimal balance between promoting neurogenesis and reducing intracellular β-amyloid accumulation.

Oligomeric Aβ species in the brain are considered the major toxic form of Aβ, and activate a detrimental cascade that leads to neuronal loss, cognitive decline, and eventually AD diagnosis [1], [27], [28], [29], [30], [31], [32]. Increasing evidence indicates that treatments initiated late in the disease process, i.e. dense and wide distribution of Aβ plaques, have a low probability of clinical success [33] suggesting that targeting the early phase of oligomeric Aβ generation may have greater clinical efficacy. Results demonstrated that APα administered 1/week/6 months significantly increased survival of newly-generated neurons and simultaneously reduced Aβ pathology in both age groups. While AP significantly reduced the 56 kD Aβ oligomer in both the pre-pathology and post-pathology groups, greatest efficacy was observed when APα was administered in the pre-pathology phase where APα reduced both the 27 kD and 56 kD Aβ oligomers. Consistent with the biochemical results, Aβ immunoreactivity was reduced in hippocampal CA1 and moderately reduced in frontal cortex, amygdala and subiculum. Aβ binding alcohol dehydrogenase, a mitochondrial Aβ binding enzyme, was reduced in parallel with Aβ by APα treatment. ABAD has been well-demonstrated to interact with Aβ within mitochondria and cause mitochondrial dysfunction [16]. In AD patients and the 3xTgAD mice, ABAD expression parallels severity of Aβ load [19]. Based on these findings, reduction of both Aβ and ABAD load with APα treatment would be predicted to prevent the exacerbation of mitochondrial deficits associated with AD and therefore delay or prevent disease progression.

In this mouse model, phosphorylated-tau follows severity of Aβ accumulation [34]. The APα-induced reduction in intraneuronal β-amyloid was accompanied by a moderate, although not statistically significant, brain-wide reduction in phosphorylated-tau with the greatest reduction of phosphorylated-tau in AD-vulnerable brain regions. The reduction of tau pathology by APα can likely be attributed to the reduction in Aβ load. Microglial activation is a well-documented response to AD pathology [35], [36], [37]. In parallel to the reduction of Aβ and phospho-tau, APα treatment significantly reduced microglial activation as indicated by reduced expression of OX42. While the reduction in microglia activation by APα is likely a consequence of APα-induced reduction of AD pathology, suppression of microglial activation would relieve the inflammatory burden associated with AD pathology.

White matter abnormalities have been widely reported in AD [2], [3], [4], [38] and observed in the 3xTgAD mouse brain as well [6]. In the 3xTgAD mouse brain, abnormal myelination and loss of axonal integrity occur in the same brain regions vulnerable to AD pathology in humans [6]. In the present study, we found that in parallel to reduction of AD pathology, APα treatment also increased a marker of myelination in these same brain regions. Collectively, results of these analyses demonstrate the concomitant reduction by APα of four major pathological markers of AD, Aβ, phospho-tau, microglial activation and white matter loss, and provide pre-clinical evidence in support of the efficacy of APα to decrease and delay development of AD pathology.

Increasing evidence indicates that altered cholesterol metabolism is linked to AD pathology [39], [40]. Mechanistic analyses indicated that 1/week APα begun at 3 months and continued for 6 months increased expression of LXR, PXR, and HMG-CoA reductase, three proteins that regulate cholesterol homeostasis. LXR, a nuclear hormone receptor abundant in the brain [41], acts as a molecular sensor of cholesterol levels and initiates cholesterol clearance [15]. LXR activation increases cholesterol efflux through up-regulating ABCA1 and ApoE expression, and prevents the hyper-activation of γ-secretase and over-production of Aβ [15], [42], [43]. LXR activation has been demonstrated to improve cognitive function in multiple mouse models of amyloidogenesis [15], [44], [45], [46], [47].

LXR ligands frequently activate PXR [48]. Results from our analyses indicated that in parallel with an APα-induced increase in LXR expression in the pre-pathology condition, APα also increased PXR expression in the pre-pathology 3xTgAD mouse brain. PXR activation induces CYP3A enzymes including CYP3A4 and CYP3A13 and leads to cholesterol hydroxylation and activation of organic anion transporters for cholesterol extrusion [49]. The APα induced increase in brain LXR and PXR leads to increased cholesterol efflux, thereby reducing γ-secretase activation by cholesterol-laden lipid rafts. Increased cholesterol efflux provides a plausible mechanism to explain how APα decreased the generation of both 27 kD and 56 kD intraneuronal Aβ oligomers.

To determine whether APα primarily effected cholesterol homeostatic mechanisms primarily in brain, we investigated LXR and PXR expression in liver. Further, these analyses would also address whether the vehicle (2-hydroxypropyl-β-cyclodextrin), which can reduce cholesterol in other tissues, would also affect expression of HMG Co-A reductase, LXR and PXR in liver. To address these issues, we conducted Western blot analysis for LXR and PXR expression in liver. Results of these analyses indicated no significant effect of APα on LXR or PXR expression in liver (data not shown). These data support our findings indicating that APα is primarily affecting cholesterol homeostasis in brain without affecting peripheral cholesterol.

These findings in the 3xTgAD mouse brain are consistent with APα induced activation of a PXR pathway in cholesterol trafficking in the Niemann-Pick C Disease mouse model [22]. APα-induced reduction of AD pathology burden is also consistent with the findings of Mellon and colleagues who reported that APα delayed progression of Niemann-Pick C Disease in a mouse model [23], [52], [53]. In young animals, either single or multiple injections of APα protected cerebellar Purkinje cells from degeneration and increased animal life span [52]. Less improvement was observed at older ages of Niemann-Pick C1−/− mouse that had disrupted neurosteroidogenesis [52]. APα induced a delay in progression of pathology and enhanced survival of Niemann-Pick C mice through a PXR-mediated mechanism [22].

Our study also revealed that APα induces an increase in HMG-CoA reductase protein expression. The increase in HMG-CoA reductase is at first paradoxical as it is the rate-limiting enzyme in cholesterol synthesis. HMG-CoA reductase is also required for oxysterol generation which is well documented to activate LXR and PXR-mediated gene transcription for cholesterol and lipid transport proteins [40]. If this hypothesis is correct, it would predict decreased activation of γ-secretase by cholesterol and lipid laden lipid rafts.

In this study, an interesting and consistent finding was that the pre-pathology treatment exhibited a greater magnitude of efficacy in terms of promoting survival of neural progenitors, reducing Aβ pathology, suppressing inflammatory response, and activating LXR/PXR pathways involved in cholesterol homeostasis and Aβ clearance. These findings indicate that early development of pathology serves as a critical stage for APα therapeutic efficacy that coincides with intraneuronal Aβ accumulation. The appearance of Aβ plaques coincides with cessation of APα efficacy. One potential reason for this cessation is that Aβ has been transported out of the cell and our data suggest that intraneuronal Aβ accumulation is a determining factor in efficacy of APα which is paralleled by the lack of efficacy once intraneuronal Aβ is extracellularly localized. The well-established relationship between cholesterol homeostasis and Aβ generation coupled with our findings of APα induced pathways of cholesterol homeostasis coinciding with intracellular Aβ, suggest that these two systems, i.e. cholesterol homeostasis and intraneuronal Aβ and APα efficacy are coupled.

The deposition of Aβ in the extracellular compartment disconnects this coupled pathway, leading to a loss of efficacy of APα. Our data indicate that the presence of intraneuronal Aβ is the key regulatory factor in determining therapeutic efficacy. Although the molecular content of extracellular and intraneuronal Aβ may be similar, their localization is a key indicator of cellular adaptation and therapeutic regulation.

### Translational implications for the optimal therapeutic regimen

APα induction of neurogenesis [7], [8], recovery of learning and memory function [8], and reduction of AD pathology burden provides pre-clinical evidence for APα as a multifaceted regenerative therapeutic. Moreover, APα induction of cell cycle gene expression [7] and key regulators of cholesterol homeostasis provides mechanistic plausibility for its therapeutic efficacy to promote neurogenesis and cognitive function while reducing AD pathology. Two factors are critical to the regimen of APα to achieve therapeutic efficacy. The first of these factors is the temporal regimen of administration. Our data show that regeneration is achieved with either 1/month or 1/week regimen of APα. Reduction of AD pathology can be achieved with 1/week or 3/week regimen. The combination of regeneration and reduction of pathology was only achievable with the 1/week APα treatment regimen. The second regulating factor is the type of pathology. Administration of APα prior to and during the early stages of AD pathology (intraneuronal Aβ) was efficacious in reducing the burden of pathology. APα treatment initiated at the stage of Aβ plaque development was associated with reduction in efficacy. These findings predict that APα therapeutic benefit in humans would be most efficacious to delay progression of AD when brains still have neurogenic and myelination capacity. Target populations could include those with very early stage genetically mediated familial AD and those diagnosed with mild cognitive impairment (MCI).

## Materials and Methods

### Animal Treatments and Ethics

All rodent experiments were performed following National Institutes of Health guidelines on use of laboratory animals and an approved protocol by the University of Southern California Institutional Animal Care and Use Committee (Protocol Number: 11156). The presented study has been approved by the University of Southern California Institutional Animal Care and Use Committee (Ethics Committee).

### Transgenic Mice

Colonies of 3xTgAD (3xTgAD, homozygous mutant of human APPswe and tauP301L and PS1M146V) and nonTg mouse strain (C57BL6/129S; Gift from Dr. Frank LaFerla, University of California, Irvine) [5] were bred and maintained at the University of Southern California (Los Angeles, CA) following National Institutes of Health guidelines on use of laboratory animals and an approved protocol by the University of Southern California Institutional Animal Care and Use Committee. In addition, the minimal number of required animals was used for these experiments and pain was minimized. Mice were housed on 12 h light/dark cycles and provided with *ad libitum* access to food and water. The characterization of amyloid and tau pathologies, as well as synaptic dysfunction in this line of mice has been described previously [20], [54] and confirmed in our laboratory. The mice were genotyped regularly to confirm the purity of the colony. To ensure the stability of AD-like phenotype in the 3xTgAD mouse colony, we performed routine immunohistological assays every three to four generations. Only offspring from breeders that exhibited stable AD pathology were randomized into the study. The number of mice per condition is indicated within the results section. Experiments were performed using three- and six-month-old male 3xTgAD and age-matched nonTg mice.

### Drug Preparation

Allopregnanolone (APα 3α-hydroxy-5α-pregnan-20-one) (aka AP, Allo or THP) used for this study was purchased from Steraloids, Inc. (Newport, Rhode Island, USA). APα was dissolved in 22.5% (W/V) (2-hydroxypropyl)-β-cyclodextrin (Sigma, St. Louis, MO) solution at APα 2.5 mg/ml by brief sonication and was subcutaneously injected to mice at APα 10 mg/kg body weight. β-cyclodextrin alone was included as vehicle control. Thymidine analogues, 5-Bromo-2′-deoxyuridine (BrdU), 5-Chloro-2′-deoxyuridine (CldU) and iodo-deoxyuridine (IdU), used in this study were purchased from Sigma. These analogues were dissolved in PBS and intraperitoneally (i.p.) injected following or during APα treatment at 100 mg/kg.

### Treatment paradigms

Three different APα treatment paradigms were adopted to determine the optimal APα treatment regimen to promote regenerative capacity and simultaneous reduce AD pathology. Details of these treatment paradigms are listed below (Figure 1A):

#### Paradigm 1

Three-month-old male 3xTgAD mice were injected with either APα (10 mg/kg) or vehicle once and analyzed 1 month later (1/month). One hour after APα injection, mice were injected with BrdU (100 mg/kg). Mice were sacrificed 27 days after APα administration for cell survival assessment.

#### Paradigm 2

APα was injected to mice once a week for 6 months (1/week/6 months). In order to investigate whether APα treatment could prevent or reverse AD-related pathologies, we chose 3- and 6-month-old 3xTgAD and age-matched nonTg mice for the experiments on our previous observation that detectable intraneuronal Aβ pathology starts at about 6 months of age in the male 3xTgAD model, whereas tau pathology follows Aβ pathology development and is detectable at older age [5]. During APα (10 mg/kg) treatment, mice were treated with sequential injections of BrdU (100 mg/kg) at the first 5 days of the study, IdU (100 mg/kg) for 5 days at the midpoint, and CldU (100 mg/kg) 5 days before completion of the study. After 6 months of treatment, mice at 9 and 12 months of age were sacrificed.

#### Paradigm 3

Three-month-old male 3xTgAD mice were injected with either APα (10 mg/kg) or vehicle once every other day for 3 months (3/week/3 months). In the first five weeks, BrdU (100 mg/kg) was injected 1 h after each APα given. One week before sacrifice, BrdU was injected again 1 h after APα injection.

### Animal Dissection and Tissue Collection

Upon completion of the treatments, animals were sacrificed. Prior to sacrifice, mice were anesthetized with 100 mg/kg ketamine and 10 mg/kg xylazine and perfused with pre-chilled PBS. Brains were immediately dissected along the sagittal line into two hemispheres; the left hemisphere was frozen on dry ice and then stored at −80°C for biochemical analysis, and the right hemisphere was post-fixed in 4% paraformaldehyde for immunohistochemical analysis. Fixed brains were embedded into blocks (40 brain hemispheres in the same block) for cryostat sectioning by NeuroScience Associates (NSA, Inc., Knoxville, TN). The 40-brain hemisphere block was serially sliced into 35 µm coronal sections and the free-floating multibrain sections were kept in antigen preservation solution (1% Polyvinyl pyrrolidone and 50% Ethylene glycol in PBS) at −20°C until use.

### Unbiased Stereology

Number of BrdU-labeled cells was determined in every sixth section in a series of 40 µm coronal sections using unbiased stereology (optical dissector). The first section of each hemisphere was randomly started at the beginning of olfactory, and serial sections were collected to the end of the cerebellum. Systematic samplings of unbiased counting frames of 50 µm on a side with a 200 µm matrix spacing were produced using a semiautomatic stereology system (Zeiss Axiovert 200 M fluorescent microscope as part of the 3iMarianas digital microscopy and a 60× SPlan apochromat oil objective (1.4 numerical aperture)). Positive cells that intersected the uppermost focal (exclusion) plane and those that intersected the exclusion boundaries of the unbiased sampling frame were excluded from analysis. Cells that met analysis criteria through a 20 µm axial distance were counted according to the optical dissector principle. The granule cell layer reference volume was determined by summing the traced SGZ, granule cell areas for each section multiplied by the distance between sections sampled. The mean granule cell number per dissector volume was multiplied by the reference volume to estimate the total granule cell number. The stereologically determined number of BrdU-positive cells was related to the granule cell layer sectional volume and multiplied by the reference volume to estimate the total number of BrdU-positive cells.

### Flow-cytometry Analysis of BrdU, IdU, and CldU Incorporation

Along with the study, our group developed a flow cytometry counting method for analysis of BrdU, IdU, and CldU incorporation. This flow cytometry analysis method has been previously validated by direct comparison between unbiased stereology and flow cytometry analysis [8], [55]. Briefly, hippocampi were dissected from the fixed hemispheres using anatomical landmarks as described [56]. Extracted hippocampi were homogenized and nuclei sample collected into a 1.5 ml microcentrifuge tube, washed four times using 200 µl of PBS, and then centrifuged for 10 min at 10,000 rpm. The pellet was then resuspended in 600 µl of PBS plus 0.5% Triton X-100, heated for 1 h at 75 C° for epitope retrieval, and incubated for 24 h at 4 C° with primary mouse monoclonal anti-BrdU antibody (1∶100, Ab12219, Abcam, Cambridge, MA) for BrdU+ cell count; anti-CldU (1∶40, ab6326, Abcam) for CldU+ cell count; anti-IdU (1∶20, BD, 340649), for IdU+ cell count. The number of nuclei was estimated by counting the propidium iodide, and the number of BrdU-, IdU- and CldU-labeled cells was detected using Beckman Flow Cytometry System (FC 500) with CXP Software.

### Immunoblotting

Protein was extracted from cerebral cortex using T-per (Pierce) extraction buffer complemented with Protease and Phosphatase Inhibitor (Sigma, St. Louis, MO), followed by high-speed centrifugation at 100,000 x *g* for 1 h. The supernatant was collected as the protein extract. Protein concentrations were determined by DC protein assay kit (Bio-Rad, Hercules, CA). Equal amounts of protein (40–60 µg depending on the protein of interest) were separated by SDS-PAGE on a 12% or 10–20% Bis-Tris gel (Bio-Rad), transferred to 0.2 µm Nitrocellulose or 0.45 µm PVDF membrane (Millipore Corp., Bedford, MA). Nonspecific binding sites were blocked with blocking buffer (5% nonfat milk in Tris-buffered saline, TBS, containing 0.1% Tween-20, TBST). After blocking, primary antibodies were incubated in blocking buffer overnight at 4°C. The following antibodies were used in this study: β-Amyloid (Aβ) monoclonal antibody, 6E10 (1∶1000, Covance/Signet, Berkeley, CA); for the abnormally processed isoforms as well as precursor forms and oligomers of β-amyloid [27]; mouse anti-human PHF-Tau clone AT8 (1∶500, Pierce) for phosphorylated tau protein at serine 202; rabbit polyclonal anti-CD11b/c (OX42, 1∶500, Neuromics) for microglial cells [57]; rabbit polyclonal to ERAB (1∶500 Abcam) for Aβ peptide binding protein on mitochondria, rabbit polyclonal anti-HMG-CoA reductase (1∶1000, Upstate); rabbit polyclonal anti-LXR β (1∶500, Novus) for Liver X Receptor (LXR); rabbit polyclonal anti-PXR (1∶500, Novus) for Pregnane X Receptor (PXR); CNPase (1∶1000, Chemicon) an oligodendrocyte marker to detect myelination [6]. The membranes were then incubated with a horseradish peroxidase-conjugated goat anti-mouse or anti-rabbit secondary antibody (1∶5,000–10,000) complementary to the primary antibody. Blots were developed with Supersignal West Dura Extended Duration Substrate (Pierce) or TMB (3, 3′, 5, 5′-Tetramethylbenzidine) Substrate kit (Vector). Results were visualized with the Chemidoc System (Bio-Rad). Quantitative analyses of optical band intensity were performed by BioRad Quantity One software or Un-Scan-It. Protein expression was normalized by loading housekeeping protein β-actin or β-tubulin.

### Immunofluorescent Labeling

Free-floating multibrain sections were rinsed extensively in PBS containing 0.1% Triton X-100 (PBST). They were blocked in blocking buffer (PBST with 5% normal goat serum, NGS, and 0.3% Triton X-100) for 60 min, followed by incubation with primary antibody in blocking buffer overnight at 4°C. The following antibodies were used: β-Amyloid (A) monoclonal antibody, 6E10 (1∶1000, Signet, Cat No. 9320), mouse anti-human PHF-Tau MAb clone AT8 (Pierce, MN1020), rabbit polyclonal anti-Iba1 (1∶1000, Wako) for microglia cells [58], goat polyclonal anti-ERAB (C-20, 1∶250, Santa Cruz) for Aβ peptide binding protein on mitochondria, CNPase (1∶300, Chemicon) an oligodendrocyte marker for labeling myelin structure of white matter in brain. The next day, sections were rinsed 3 times with PBST and incubated in FITC-conjugated goat anti-mouse/rabbit (1∶500, Vector Laboratories) or Cy3-conjugated goat anti-mouse/rabbit (1∶1000, GE Healthcare) secondary antibody in PBST for 60 min at room temperature. After intense washing with PBST the sections were mounted with DAPI-containing mounting medium on coverslips. The immunoreactivity was observed and images were captured with the Axiovert 200 M Marianas Digital Microscopy Workstation (Intelligent Imaging Innovations, Denver, CO).

### Statistical Analysis

Statistical significance for group comparison was performed by a Student's t-Test or one way ANOVA followed by a Newman-Keuls post-hoc analysis. The difference between groups was considered significant when the *P* value was <0.05.

## References

[1] Selkoe DJ (2001). Alzheimer's disease results from the cerebral accumulation and cytotoxicity of amyloid beta-protein.. J Alzheimers Dis.

[2] Bartzokis G (2004). Age-related myelin breakdown: a developmental model of cognitive decline and Alzheimer's disease.. Neurobiol Aging.

[3] Bartzokis G, Cummings JL, Sultzer D, Henderson VW, Nuechterlein KH (2003). White matter structural integrity in healthy aging adults and patients with Alzheimer disease: a magnetic resonance imaging study.. Arch Neurol.

[4] Bartzokis G, Lu PH, Tingus K, Mendez MF, Richard A (2010). Lifespan trajectory of myelin integrity and maximum motor speed.. Neurobiol Aging.

[5] Oddo S, Caccamo A, Shepherd JD, Murphy MP, Golde TE (2003). Triple-transgenic model of Alzheimer's disease with plaques and tangles: intracellular Abeta and synaptic dysfunction.. Neuron.

[6] Desai MK, Sudol KL, Janelsins MC, Mastrangelo MA, Frazer ME (2009). Triple-transgenic Alzheimer's disease mice exhibit region-specific abnormalities in brain myelination patterns prior to appearance of amyloid and tau pathology.. Glia.

[7] Wang JM, Johnston PB, Ball BG, Brinton RD (2005). The neurosteroid allopregnanolone promotes proliferation of rodent and human neural progenitor cells and regulates cell-cycle gene and protein expression.. J Neurosci.

[8] Wang JM, Singh C, Liu L, Irwin RW, Chen S (2010). Allopregnanolone reverses neurogenic and cognitive deficits in mouse model of Alzheimer's disease.. Proc Natl Acad Sci U S A.

[9] Marx CE, Trost WT, Shampine LJ, Stevens RD, Hulette CM (2006). The Neurosteroid Allopregnanolone Is Reduced in Prefrontal Cortex in Alzheimer's Disease.. Biol Psychiatry.

[10] Naylor JC, Kilts JD, Hulette CM, Steffens DC, Blazer DG (2010). Allopregnanolone levels are reduced in temporal cortex in patients with Alzheimer's disease compared to cognitively intact control subjects.. Biochim Biophys Acta.

[11] Schumacher M, Guennoun R, Robert F, Carelli C, Gago N (2004). Local synthesis and dual actions of progesterone in the nervous system: neuroprotection and myelination.. Growth Hormone & Igf Research.

[12] Mellon SH, Gong W, Schonemann MD (2008). Endogenous and synthetic neurosteroids in treatment of Niemann-Pick Type C disease.. Brain Res Rev.

[13] Oosterveer MH, Grefhorst A, Boesjes M, Bos T, Kuipers F (2010). Time-dependent effects of pharmacological Liver X Receptor activation in mice.. European Journal of Clinical Investigation.

[14] Zhou H, Li Z, Silver DL, Jiang XC (2006). Cholesteryl ester transfer protein (CETP) expression enhances HDL cholesteryl ester liver delivery, which is independent of scavenger receptor BI, LDL receptor related protein and possibly LDL receptor.. Biochim Biophys Acta.

[15] Koldamova RP, Lefterov IM, Staufenbiel M, Wolfe D, Huang S (2005). The liver X receptor ligand T0901317 decreases amyloid beta production in vitro and in a mouse model of Alzheimer's disease.. J Biol Chem.

[16] Lustbader JW, Cirilli M, Lin C, Xu HW, Takuma K (2004). ABAD directly links Abeta to mitochondrial toxicity in Alzheimer's disease.. Science.

[17] Takuma K, Yao J, Huang J, Xu H, Chen X (2005). ABAD enhances Abeta-induced cell stress via mitochondrial dysfunction.. FASEB J.

[18] Yan SD, Stern DM (2005). Mitochondrial dysfunction and Alzheimer's disease: role of amyloid-beta peptide alcohol dehydrogenase (ABAD).. Int J Exp Pathol.

[19] Yao J, Irwin RW, Zhao L, Nilsen J, Hamilton RT (2009). Mitochondrial bioenergetic deficit precedes Alzheimer's pathology in female mouse model of Alzheimer's disease.. Proc Natl Acad Sci U S A.

[20] Oddo S, Caccamo A, Kitazawa M, Tseng BP, LaFerla FM (2003). Amyloid deposition precedes tangle formation in a triple transgenic model of Alzheimer's disease.. Neurobiol Aging.

[21] Lamba V, Yasuda K, Lamba JK, Assem M, Davila J (2004). PXR (NR1I2): splice variants in human tissues, including brain, and identification of neurosteroids and nicotine as PXR activators.. Toxicol Appl Pharmacol.

[22] Langmade SJ, Gale SE, Frolov A, Mohri I, Suzuki K (2006). Pregnane X receptor (PXR) activation: a mechanism for neuroprotection in a mouse model of Niemann-Pick C disease.. Proc Natl Acad Sci U S A.

[23] Ahmad I, Lope-Piedrafita S, Bi X, Hicks C, Yao Y (2005). Allopregnanolone treatment, both as a single injection or repetitively, delays demyelination and enhances survival of Niemann-Pick C mice.. J Neurosci Res.

[24] Jacobs HI, Visser PJ, Van Boxtel MP, Frisoni GB, Tsolaki M (2010). The association between white matter hyperintensities and executive decline in mild cognitive impairment is network dependent.. Neurobiol Aging. [Aug 23, Epub ahead of print] PMID:.

[25] Kuczynski B, Targan E, Madison C, Weiner M, Zhang Y (2010). White matter integrity and cortical metabolic associations in aging and dementia.. Alzheimers Dement.

[26] Singh C, Liu L, Wang JM, Irwin RW, Yao J (2011). Allopregnanolone restores hippocampal-dependent learning and memory and enhances neural progenitor survival in aging 3xTgAD and nonTg mice.. Neurobiol Aging. [July 29, Epub ahead of print] PMID:.

[27] Lesne S, Koh MT, Kotilinek L, Kayed R, Glabe CG (2006). A specific amyloid-beta protein assembly in the brain impairs memory.. Nature.

[28] Oda T, Wals P, Osterburg HH, Johnson SA, Pasinetti GM (1995). Clusterin (apoJ) alters the aggregation of amyloid beta-peptide (A beta 1–42) and forms slowly sedimenting A beta complexes that cause oxidative stress.. Exp Neurol.

[29] Lambert MP, Barlow AK, Chromy BA, Edwards C, Freed R (1998). Diffusible, nonfibrillar ligands derived from Abeta1–42 are potent central nervous system neurotoxins.. Proc Natl Acad Sci U S A.

[30] Klyubin I, Walsh DM, Lemere CA, Cullen WK, Shankar GM (2005). Amyloid beta protein immunotherapy neutralizes Abeta oligomers that disrupt synaptic plasticity in vivo.. Nat Med.

[31] Klein WL, Krafft GA, Finch CE (2001). Targeting small Abeta oligomers: the solution to an Alzheimer's disease conundrum?. Trends Neurosci.

[32] Klein WL, Stine WB, Teplow DB (2004). Small assemblies of unmodified amyloid beta-protein are the proximate neurotoxin in Alzheimer's disease.. Neurobiol Aging.

[33] Brinton RD (2008). The healthy cell bias of estrogen action: mitochondrial bioenergetics and neurological implications.. Trends Neurosci.

[34] Oddo S, Billings L, Kesslak JP, Cribbs DH, LaFerla FM (2004). Abeta immunotherapy leads to clearance of early, but not late, hyperphosphorylated tau aggregates via the proteasome.. Neuron.

[35] Li R, Shen Y, Yang LB, Lue LF, Finch C (2000). Estrogen enhances uptake of amyloid beta-protein by microglia derived from the human cortex.. J Neurochem.

[36] Xie Z, Harris-White ME, Wals PA, Frautschy SA, Finch CE (2005). Apolipoprotein J (clusterin) activates rodent microglia in vivo and in vitro.. J Neurochem.

[37] Xie Z, Wei M, Morgan TE, Fabrizio P, Han D (2002). Peroxynitrite mediates neurotoxicity of amyloid beta-peptide1–42- and lipopolysaccharide-activated microglia.. J Neurosci.

[38] Kuczynski B, Reed B, Mungas D, Weiner M, Chui HC (2008). Cognitive and anatomic contributions of metabolic decline in Alzheimer disease and cerebrovascular disease.. Archives of Neurology.

[39] Repa JJ, Li H, Frank-Cannon TC, Valasek MA, Turley SD (2007). Liver X receptor activation enhances cholesterol loss from the brain, decreases neuroinflammation, and increases survival of the NPC1 mouse.. J Neurosci.

[40] Leduc V, Jasmin-Belanger S, Poirier J (2010). APOE and cholesterol homeostasis in Alzheimer's disease..

[41] Whitney KD, Watson MA, Collins JL, Benson WG, Stone TM (2002). Regulation of cholesterol homeostasis by the liver X receptors in the central nervous system.. Mol Endocrinol.

[42] Xiong H, Callaghan D, Jones A, Walker DG, Lue LF (2008). Cholesterol retention in Alzheimer's brain is responsible for high beta- and gamma-secretase activities and Abeta production.. Neurobiol Dis.

[43] Schultz JR, Tu H, Luk A, Repa JJ, Medina JC (2000). Role of LXRs in control of lipogenesis.. Genes Dev.

[44] Donkin JJ, Stukas S, Hirsch-Reinshagen V, Namjoshi D, Wilkinson A (2010). ATP-binding cassette transporter A1 mediates the beneficial effects of the liver-X-receptor agonist GW3965 on object recognition memory and amyloid burden in APP/PS1 mice.. J Biol Chem.

[45] Jiang Q, Lee CY, Mandrekar S, Wilkinson B, Cramer P (2008). ApoE promotes the proteolytic degradation of Abeta.. Neuron.

[46] Sun Y, Yao J, Kim TW, Tall AR (2003). Expression of liver X receptor target genes decreases cellular amyloid beta peptide secretion.. J Biol Chem.

[47] Riddell DR, Zhou H, Comery TA, Kouranova E, Lo CF (2007). The LXR agonist TO901317 selectively lowers hippocampal Abeta42 and improves memory in the Tg2576 mouse model of Alzheimer's disease.. Mol Cell Neurosci.

[48] Shenoy SD, Spencer TA, Mercer-Haines NA, Alipour M, Gargano MD (2004). CYP3A induction by liver x receptor ligands in primary cultured rat and mouse hepatocytes is mediated by the pregnane X receptor.. Drug Metab Dispos.

[49] Sonoda J, Xie W, Rosenfeld JM, Barwick JL, Guzelian PS (2002). Regulation of a xenobiotic sulfonation cascade by nuclear pregnane X receptor (PXR).. Proc Natl Acad Sci U S A.

[50] Davidson CD, Ali NF, Micsenyi MC, Stephney G, Renault S (2009). Chronic cyclodextrin treatment of murine Niemann-Pick C disease ameliorates neuronal cholesterol and glycosphingolipid storage and disease progression.. PLoS One.

[51] Rosenbaum AI, Zhang G, Warren JD, Maxfield FR (2010). Endocytosis of beta-cyclodextrins is responsible for cholesterol reduction in Niemann-Pick type C mutant cells.. Proc Natl Acad Sci U S A.

[52] Griffin LD, Gong W, Verot L, Mellon SH (2004). Niemann-Pick type C disease involves disrupted neurosteroidogenesis and responds to allopregnanolone.. Nat Med.

[53] Mellon S, Gong W, Griffin LD (2004). Niemann pick type C disease as a model for defects in neurosteroidogenesis.. Endocr Res.

[54] Billings LM, Oddo S, Green KN, McGaugh JL, LaFerla FM (2005). Intraneuronal Abeta causes the onset of early Alzheimer's disease-related cognitive deficits in transgenic mice.. Neuron.

[55] Henry S, Bigler S, Wang J (2009). High throughput analysis of neural progenitor cell proliferation in adult rodent hippocampus.. Biosci Trends.

[56] Bilsland JG, Haldon C, Goddard J, Oliver K, Murray F (2006). A rapid method for the quantification of mouse hippocampal neurogenesis in vivo by flow cytometry. Validation with conventional and enhanced immunohistochemical methods.. J Neurosci Methods.

[57] Mika J, Osikowicz M, Rojewska E, Korostynski M, Wawrzczak-Bargiela A (2009). Differential activation of spinal microglial and astroglial cells in a mouse model of peripheral neuropathic pain.. Eur J Pharmacol.

[58] Ahmed Z, Shaw G, Sharma VP, Yang C, McGowan E (2007). Actin-binding proteins coronin-1a and IBA-1 are effective microglial markers for immunohistochemistry.. J Histochem Cytochem.

